# Increased angiogenic factor secretion by decidual natural killer cells from pregnancies with high uterine artery resistance alters trophoblast function

**DOI:** 10.1093/humrep/deu017

**Published:** 2014-02-12

**Authors:** A.E. Wallace, R. Fraser, S. Gurung, S.S. Goulwara, G.S. Whitley, A.P. Johnstone, J.E. Cartwright

**Affiliations:** Division of Biomedical Sciences, St. George's, University of London, Cranmer Terrace, LondonSW17 ORE, UK

**Keywords:** natural killer cell, angiogenin, endostatin, pre-eclampsia, decidua

## Abstract

**STUDY QUESTION:**

Are the concentrations of factors secreted by decidual natural killer (dNK) cells from pregnancies at high risk of poor spiral artery remodelling different to those secreted from pregnancies at low risk?

**SUMMARY ANSWER:**

Expression levels of PLGF, sIL-2R, endostatin and angiogenin were significantly increased by dNK cells from high-risk pregnancies, and angiogenin and endostatin were found to alter trophoblast function.

**WHAT IS KNOWN ALREADY:**

During early pregnancy, maternal uterine spiral arteries are remodelled from small diameter, low-flow, high-resistance vessels into larger diameter, higher flow vessels, with low-resistance. This change is essential for the developing fetus to obtain sufficient oxygen and nutrients. dNK cells have been implicated in this process.

**STUDY DESIGN, SIZE, DURATION:**

dNK cells were isolated from first trimester terminations of pregnancies (obtained with local ethical approval) screened for normal- or high-resistance index, indicative of cases least (<1%) and most (>21%) likely to have developed pre-eclampsia had the pregnancy not been terminated (*n* = 18 each group). Secreted factors and the effects of these on the trophoblast cell line, SGHPL-4, were assessed *in vitro*.

**PARTICIPANTS/MATERIALS, SETTING, METHODS:**

A multiplex assay was used to assess dNK cell-secreted factors. SGHPL-4 cell functions were assessed using time-lapse microscopy, 3D invasion assays, endothelial-like tube formation ability and western blot analysis.

**MAIN RESULTS AND THE ROLE OF CHANCE:**

The expression levels of PLGF (*P* < 0.01), sIL-2R (*P* < 0.01), endostatin (*P* < 0.05) and angiogenin (*P* < 0.05) were significantly increased by dNK cells from high-risk pregnancies. Endostatin significantly decreased SGHPL-4 invasion (*P* < 0.05), SGHPL-4 tube formation (*P* < 0.05) and SGHPL-4 Akt^ser473^ phosphorylation (*P* < 0.05). Angiogenin significantly decreased SGHPL-4 invasion (*P* < 0.05), but increased SGHPL-4 tube formation (*P* < 0.01) and decreased SGHPL-4 Akt^ser473^ phosphorylation (*P* < 0.05).

**LIMITATIONS, REASONS FOR CAUTION:**

The culture of dNK cells and protein concentrations *in vitro* may not fully represent the *in vivo* situation. Although SGHPL-4 cells are extravillous trophoblast derived, further studies would be needed to confirm the roles of angiogenin and endostatin *in vivo*.

**WIDER IMPLICATIONS OF THE FINDINGS:**

The altered expression of secreted factors of dNK cells may contribute to pregnancy disorders associated with poor spiral artery remodelling.

**STUDY FUNDING/COMPETING INTEREST(S):**

This study was supported by the Wellcome Trust (project reference 091550). R.F. was a recipient of a PhD studentship from the Division of Biomedical Sciences, St. George's, University of London. The authors have no conflict of interests.

## Introduction

The placental cells arising from the outer layer of the blastocyst, the trophoblast, differentiate along either the villous or extravillous pathways. Extravillous trophoblast (EVT), invade into the pregnant uterus (decidua) where they interact with maternal cells, including decidual natural killer (dNK) cells. These comprise ∼70% of the decidual immune cell population and are a distinct subset of NK cells. They display a large granular lymphocyte morphology and are CD56^bright^CD16^−^, as opposed to peripheral blood natural killer cells, where the predominant population is CD56^dim/−^CD16^bright^ (King *et al.*, 1998). During early pregnancy, maternal uterine spiral arteries are remodelled from low-flow, high-resistance vessels into higher flow vessels with low-resistance. The extent of EVT invasion is critical for implantation and remodelling of the uterine spiral arteries (Pijnenborg *et al.*, 2006), and EVT have been shown to play an active role in regulating the remodelling events (Ashton *et al.*, 2005; Keogh *et al.*, 2007; Harris *et al.*, 2010). However, there is now considerable interest in the role that other cells, particularly the dNK cells, may have in regulating trophoblast invasion.

dNK cells produce a number of soluble factors such as cytokines, growth factors and pro-and anti-angiogenic proteins, in contrast to peripheral blood NK cells, which have a more cytotoxic role in defence (Santoni *et al.*, 2007). Trophoblast invasion and spiral artery remodelling are complex processes, with many interactions taking place between the various cell types in the decidual environment. The appropriate regulation of these processes is likely to be influenced by the levels of dNK cell-derived factors since they are located in close proximity to both invading EVT and remodelling vessels and several dNK-derived cytokines, chemokines and growth factors have been identified at the fetal–maternal interface (Hanna *et al.*, 2006; Lash *et al.*, 2006). For example, interferon (IFN)-γ may modulate both chemokine expression and trophoblast activity to limit invasion (Murphy *et al.*, 2009).

In pregnancies complicated by pre-eclampsia and intrauterine growth restriction, shallow trophoblast invasion and insufficient spiral artery remodelling have been observed (Brosens *et al.*, 1970; Pijnenborg *et al.*, 1991; Meekins *et al.*, 1994). Poor spiral artery remodelling is established in the first trimester of pregnancy. However, human studies are restricted by a lack of access to first trimester tissue with a known pregnancy outcome or a known stage of spiral artery remodelling. Doppler ultrasound scanning can be used to measure the resistance index (RI) of uterine artery blood flow, reflecting the level of remodelling in the spiral arteries, and therefore can be used as a proxy measure of the remodelling process. In this study, we have used this technique to identify pregnancies at the highest risk (21%) and at lowest risk (<1%) of developing pre-eclampsia (Prefumo *et al.*, 2004), with the highest and lowest evidence of first trimester poor spiral artery remodelling.

In this study, the factors produced by isolated dNK cells were examined and the role that two of these factors, angiogenin and endostatin, may play in modulation of trophoblast activity during pregnancy was investigated.

## Materials and Methods

### Doppler ultrasound of uterine artery resistance and ethical approval

Maternal uterine artery Doppler ultrasound scans were conducted on women attending a clinic for elective termination of pregnancy as previously described (Melchiorre *et al.*, 2008; Prefumo *et al.*, 2008). Wandsworth Local Research Ethics Committee approval was in place for both the Doppler ultrasound before surgical termination and the use of first trimester tissue after the termination, and all women gave informed written consent. Terminations of pregnancy were carried out at 9–14 weeks of gestation. All were singleton pregnancies, with no known pre-existing medical conditions. High-RI cases were defined as those presenting with bilateral uterine diastolic notches and a mean RI above the 95th percentile, while normal-RI cases were defined as presenting with no diastolic notches and a mean RI below the 95th percentile. These two RI groups represent cases with the most (21%) and least (<1%) likely chance of developing pre-eclampsia, had the pregnancy not been terminated (Melchiorre *et al.*, 2008).

### Positive selection of dNK cells

Products of conception were obtained immediately after surgical termination of pregnancy. NK cells were isolated from decidual tissue using positive selection with anti-CD56 antibody coated magnetic beads (Miltenyi Biotec, Surrey, UK) as previously described (Fraser *et al.*, 2012). Purity was 93.6 ± 1.3% (mean ± SEM, *n* = 19 patients).

### Cell culture

The well-characterized human EVT cell line, SGHPL-4, is derived from primary human ﬁrst trimester EVT (Choy and Manyonda, 1998; Cartwright *et al.*, 1999). SGHPL-4 cells were cultured in Hams F10 media supplemented with 10% (v/v) fetal bovine serum (FBS), containing l-glutamine (2 mmol/l), penicillin (100 IU/ml) and streptomycin (100 µg/ml). All cells were incubated with 95% air and 5% CO_2_ at 37°C in a humidified incubator.

The isolated CD56^+^ NK cells were centrifuged at 400*g* for 10 min at 22°C and cultured for 24 h at 6 × 10^6^cells/10 ml in RPMI 1640 medium (Invitrogen, Paisley, UK) with 10% FBS, containing 2.5 µg/ml amphotericin B (Sigma Aldrich, Dorset, UK), 2 mM l-glutamine, 50 µg/ml penicillin and 50 µg/ml streptomycin (Invitrogen), 50 ng/ml stem cell factor and 5 ng/ml IL-15 (Peprotech, London, UK) at 37°C in a 5% CO_2_ humidified incubator. There was no significant difference between the gestational ages of the patients in either of the two groups (*P* = 0.235, high-RI group: *n* = at least 18 per test, mean gestational age 74.8 ± 2.4, normal-RI group: *n* = at least 18 per test, mean gestational age 77.48 ± 1.87).

### Multiplex array

Factors secreted by dNK cells were quantitatively analysed by bead-based multiplexing [angiogenin, endostatin, placental growth factor (PLGF); R&D Systems, Abingdon, UK, all other factors; Invitrogen, Life Technologies Ltd] according to manufacturer's protocols with detection on a Luminex 100 system (Luminex, Austin, TX, USA). Culture supernatants from dNK cells isolated from individual patients were examined (*n* = at least 18 normal- and high-RI samples per test). Supernatants were tested at three concentrations; concentrated 5-fold (Vivaspin columns, Sartorius Stedim UK Ltd, Surrey, UK), neat and diluted 3-fold in serum-free medium. Results were corrected according to the cellular protein concentration determined by Bradford assay (Bio-Rad, Hemel Hempstead, UK) of the pelleted cells after collection of the culture supernatant. In the case of a factor being undetected in >15% of the culture supernatants, this factor was reported but not included in the analysis. Statistical comparisons were made between the patient groups for the remaining factors.

### Motility assay

SGHPL-4 motility in response to endostatin and angiogenin was assessed as previously described using time-lapse microscopy (Grant *et al.*, 2012). SGHPL-4 cells were seeded in Hams F10 media supplemented with 10% (v/v) FBS before overnight incubation in Hams F10 media supplemented with 0.5% (v/v) FBS. Recombinant human endostatin (Peprotech) was incubated with the SGHPL-4 cells in serum-free media alone or in the presence of 10 ng/ml epidermal growth factor (EGF) at concentrations of 50, 500 and 5000 ng/ml for 24 h (*n* = 4); angiogenin (R&D Systems) was incubated with the SGHPL-4 cells alone or in the presence of 10 ng/ml EGF at concentrations of 10, 100 and 1000 ng/ml for 24 h (*n* = 4). Cells were randomly chosen at the beginning of the experimental sequence and their movement was tracked manually using Image-J software (version 1.47d, National Institutes of Health, USA).

### Invasion assay

Invasion of SGHPL-4 cells in response to recombinant endostatin and angiogenin was measured using a spheroid invasion assay as previously described (Wallace *et al.*, 2013). A volume of 100 µl of control medium or recombinant endostatin at 50, 500 and 500 ng/ml (*n* = 4) or angiogenin at 10, 100 and 1000 ng/ml (*n* = 4), with or without 10 ng/ml EGF, was added in serum-free media and spheroids were visualized after 24 h incubation using an Olympus 1X70 inverted microscope. Images were captured using a Hamamatsu C4742-95 digital camera. Invasion was measured as the average number and length of all invasive processes from each spheroid using Image-J software (version 1.47d).

### Tube formation assay

The ability of SGHPL-4 cells to form endothelial-like tube structures on Matrigel (BD, Oxford, UK) in serum-free media in the presence of angiogenin (10, 100 or 1000 ng/ml, *n* = 3) or endostatin (50, 500 or 5000 ng/ml, *n* = 5) was assessed using a µ-slide Angiogenesis Assay (Ibidi, Planegg, Germany) according to the manufacturers' instructions.

### Western blot analysis

SGHPL-4 cells were serum starved for 24 h in Hams F10 containing 0.5% FBS (v/v) before incubation of cells with 100 ng/ml angiogenin (*n* = 4) or 500 ng/ml endostatin (*n* = 4) for 0, 5, 15, 30 or 60 min in Hams F10 containing 0% FBS (v/v). SGHPL-4 cells were then lysed in Radio-Immunoprecipitation Assay buffer containing a protease inhibitor cocktail of aprotinin (60 µg/ml), phenylmethylsulfonyl fluoride (1 mM) and sodium orthovanadate (1 mM). Western blotting was then performed as previously described (Grant *et al.*, 2012) using rabbit anti-phospho-Akt^Ser473^ (1/2500 dilution, Cell Signalling Technology, Hertfordshire, UK), rabbit anti-phospho-ERK 1/2 (1/1000 dilution, Cell Signalling Technology), mouse anti-phospho-FAK (pY937, BD Biosciences, Oxford, UK) or mouse anti-α-tubulin (1/10 000 dilution, Abcam, Cambridge, UK). Western blots were scanned and the integrated density of each band determined using Image-J software (version 1.47d). Results are expressed as a ratio to the loading control within the same sample.

### Statistical analysis

Data were log transformed to stabilize variance and a Student's *t*-test was used to compare differences between patient groups using GraphPad Prism v5 (GraphPad Software, San Diego, CA, USA). A *P*-value of <0.05 was considered to be statistically significant. Non-parametric Freidman one-way analysis of variance with Dunns *post hoc* test was used to compare trophoblast invasion, tube formation and alterations in phosphorylation of signalling proteins.

## Results

### Levels of angiogenic factors in normal-RI and high-RI dNK cell conditioned media

**Figure 1:**
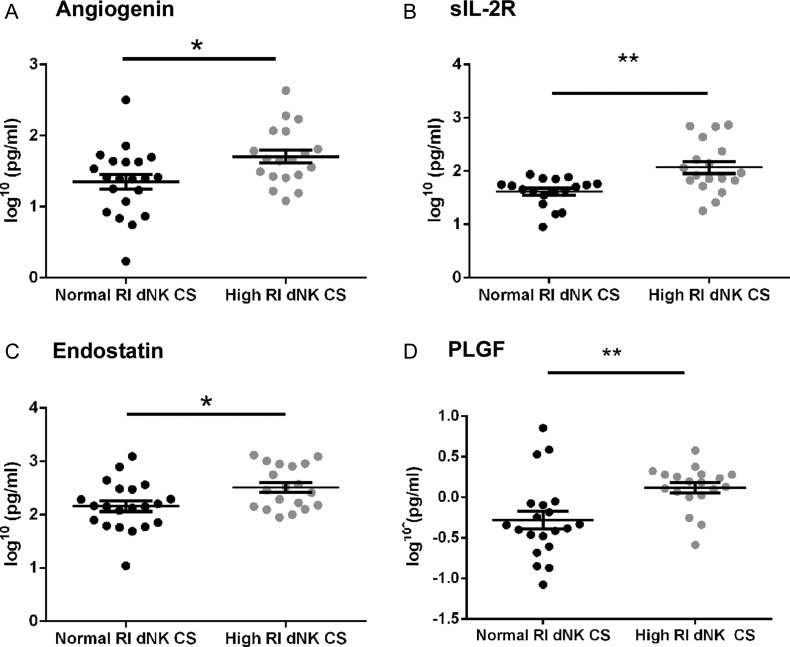
Comparison of factors secreted by normal-RI dNK cells and high-RI dNK cells. Measurement by multiplex assay of (**A**) Angiogenin (* *P* < 0.05) (**B**) sIL-2R (**P < 0.01), (**C**) Endostatin (*P < 0.05) and (**D**) PLGF (**P < 0.01) in dNK cell culture supernatants incubated for 24 h. N = at least 18 normal and high-RI samples. Data points presented are from individual patients and include the mean ± SEM. RI, resistance index.

dNK cell culture supernatants from individual patients were examined after 24 h of culture, by multiplex bead-based assays. Analysis of the various cytokines showed a significantly higher production of angiogenin (*P* < 0.05; Fig. 1A), soluble interleukin-2 receptor (sIL-2R, *P* < 0.01; Fig. 1B), endostatin (*P* < 0.05; Fig. 1C) and PLGF (*P* < 0.01; Fig. 1D) by high-RI dNK cells in comparison with normal-RI dNK cells. The following cytokines were detectable in >85% of the samples assayed, however, were not significantly different between the high-RI and normal-RI dNK groups (*P* > 0.05, data not shown): IL-1RA, monokine induced by gamma interferon (MIG), macrophage inflammatory protein 1α (MIP1α), MIP1β and regulated upon activation normal T-cell expressed and secreted (RANTES).

Several secreted factors were only detected in <15% of the total samples, and these were: fibroblast growth factor-β, granulocyte colony stimulating factor, granulocyte and macrophage colony stimulating factor, IFN-α, IFN-γ, IL-1β, IL-2, IL-7, hepatocyte growth factor, IL-10, IL-12, tumour necrosis factor α and vascular endothelial growth factor (VEGF). The factors, EGF, eotaxin, IL-4, IL-5, IL-13 and IL-17 were not detectable in any culture supernatants. When regression analysis was carried out across gestation for angiogenin, sIL-2R, endostatin, PLGF, IL-1RA, MIG, MIP1α, MIP1β and RANTES, there was no relationship between the levels of factors detected and gestational age (in either high-RI or normal-RI groups, data not shown).

### Effects of endostatin on trophoblast function

**Figure 2:**
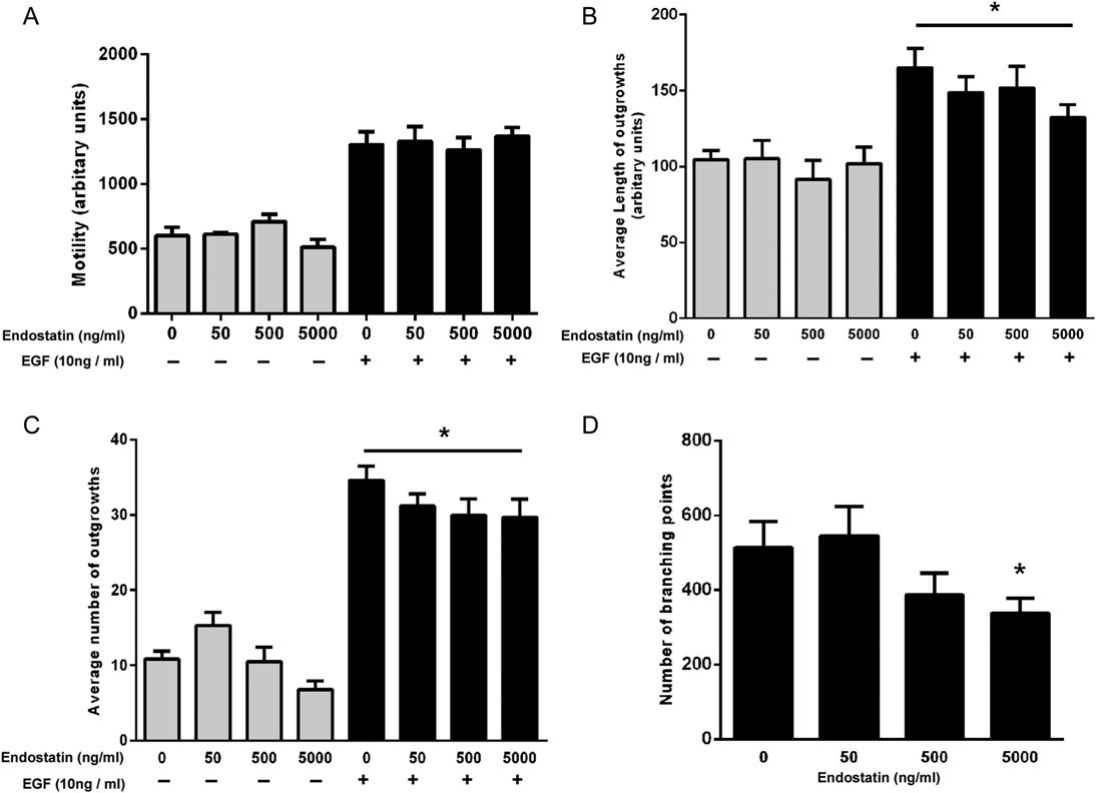
The effect of endostatin on basal and EGF-stimulated trophoblast function. Recombinant endostatin was added to SGHPL-4 cells at the concentrations listed. (**A**) SGHPL-4 cell basal and EGF-stimulated motility was measured over a 24 h period in the presence of recombinant human endostatin. Motility was not significantly different compared with the control, *n* = 4. (**B**) SGHPL-4 cells were cultured to form spheroids, embedded in fibrin gels and the length and (**C**) the number of invasive processes was measured. The average length and number of invasive process outgrowths of SGHPL-4 cell spheroids in the presence of 10 ng/ml EGF were decreased in response to 5000 ng/ml endostatin when compared with control culture media (*P < 0.05, *n* = 4). (**D**) SGHPL-4 cells were cultured on Matrigel to induce endothelial-like tube formation, which was assessed by counting the number of branching points between tubes. The total number of branching points was decreased in response to 5000 ng/ml endostatin when compared with control culture media (*P < 0.05, *n* = 5).

To transform spiral arteries, trophoblast must perform the varied functions of invasion and migration into the decidua and the transformation into an endothelial-like phenotype, and these functions may be altered by secreted factors from dNK cells. We measured the effect of recombinant endostatin on known trophoblast functions using the extravillous-like trophoblast cell line, SGHPL-4. Recombinant endostatin at concentrations of 50, 500 and 5000 ng/ml did not significantly affect basal or EGF-induced motility of SGHPL-4 cells (Fig. 2A).

Using a fibrin gel invasion assay, we investigated both the number of SGHPL-4 cells invading from a 3D spheroid and the average length of the invasive process. EGF-induced invasion of SGHPL-4 cells was significantly decreased in the presence of 5000 ng/ml endostatin, as measured by both the average length of the invasive process (Fig. 2B, control mean length: 165.2 ± 12.6, 5000 ng/ml mean length: 132.4 ± 8.4, *P* < 0.05) and number of cells invading (Fig. 2C, control mean number: 34.6 ± 1.8, 5000 ng/ml mean number: 24.6 ± 2.2, *P* < 0.05).

The ability of SGHPL-4 to form endothelial-like tube structures on Matrigel was also examined in the presence of endostatin. Endostatin at a concentration of 5000 ng/ml significantly decreased the number of branching points between trophoblast tubes (Fig. 2D, control mean no. branching points 513.5 ± 70.2, 5000 ng/ml mean no. branching points 336.8 ± 40.8, *P* < 0.05).

### Effect of angiogenin on trophoblast function

**Figure 3:**
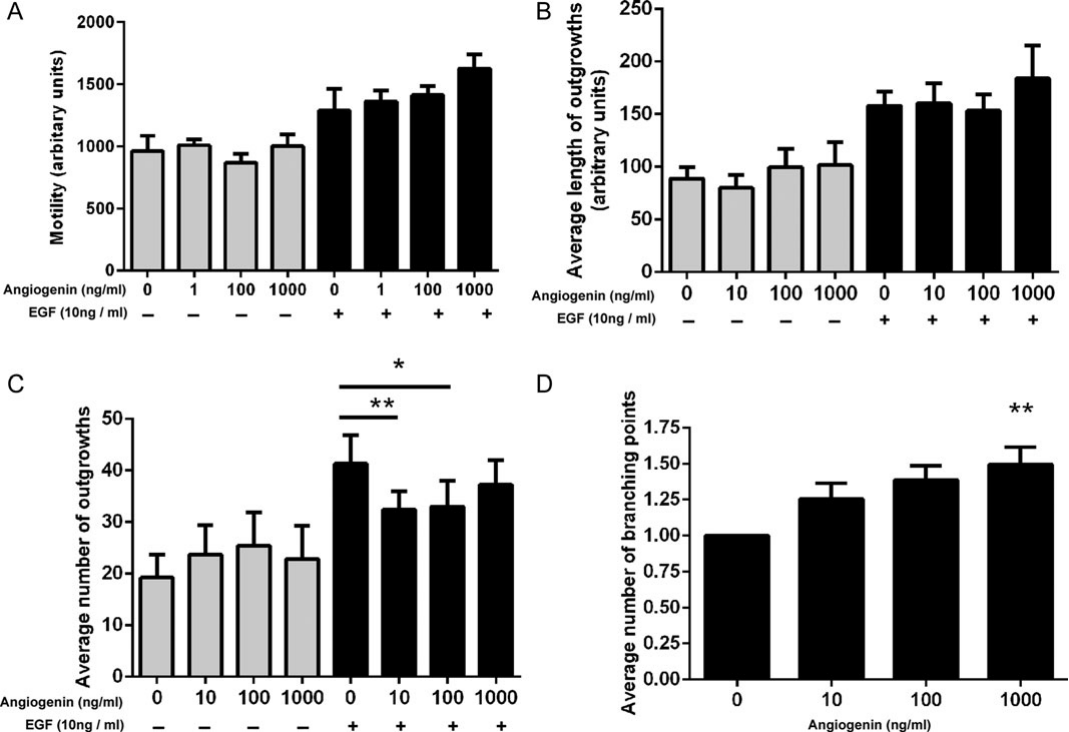
The effect of angiogenin on basal and EGF-stimulated trophoblast function. Recombinant angiogenin was added to SGHPL4 cells at the concentrations listed. (**A**) SGHPL-4 cell basal and EGF-stimulated motility was measured over a 24-h period in the presence of recombinant human angiogenin. Motility was not significantly different compared with the control, *n* = 4. (**B**) SGHPL-4 cells were cultured to form spheroids, embedded in fibrin gels and the length and (**C**) number of invasive processes was measured. The average length of invasive process outgrowths of SGHPL-4 cell spheroids alone or in the presence of 10 ng/ml EGF was not altered in response to angiogenin when compared with control culture media. The average number of invasive process outgrowths of SGHPL-4 cell by spheroids in the presence of 10 ng/ml EGF was significantly decreased in response to 10 ng/ml and 100 ng/ml angiogenin when compared with control culture media (*P < 0.05, ** *P* < 0.01, *n* = 5). (**D**) SGHPL-4 cells were cultured on reduced-growth-factor Matrigel to induce endothelial-like tube formation, assessed by counting the number of branching points between tubes. The total number of branching points was increased in response to 1000 ng/ml angiogenin when compared with control culture media (**P < 0.01, *n* = 3).

To examine the importance of increased angiogenin expression by dNK cells from high-RI patients, we measured the effect of angiogenin on the same trophoblast parameters. Recombinant angiogenin at concentrations of 10, 100 and 1000 ng/ml did not significantly affect basal or EGF-induced motility of SGHPL-4 cells (Fig. 3A). Recombinant angiogenin at concentrations of 10, 100 and 1000 ng/ml did not significantly affect the length of SGHPL-4 cell invasive processes into fibrin gels (Fig. 3B), however, at concentrations of 10 and 100 ng/ml, angiogenin did significantly inhibit the number of EGF-induced invasive outgrowths, while 1000 ng/ml did not (Fig. 3C, control mean number: 41.3 ± 5.5, 10 ng/ml mean number: 32.4 ± 3.5, 100 ng/ml mean number: 33.0 ± 4.9, *P* < 0.05). At a concentration of 1000 ng/ml, recombinant angiogenin significantly increased the number of branching points between SGHPL-4 tube-like structures formed on Matrigel (Fig. 3D, control no. branching points: 160.3 ± 52.3, 1000 ng/ml no. branching points 224 ± 62, *P* < 0.05).

### Signalling pathways affected by endostatin and angiogenin

**Figure 4:**
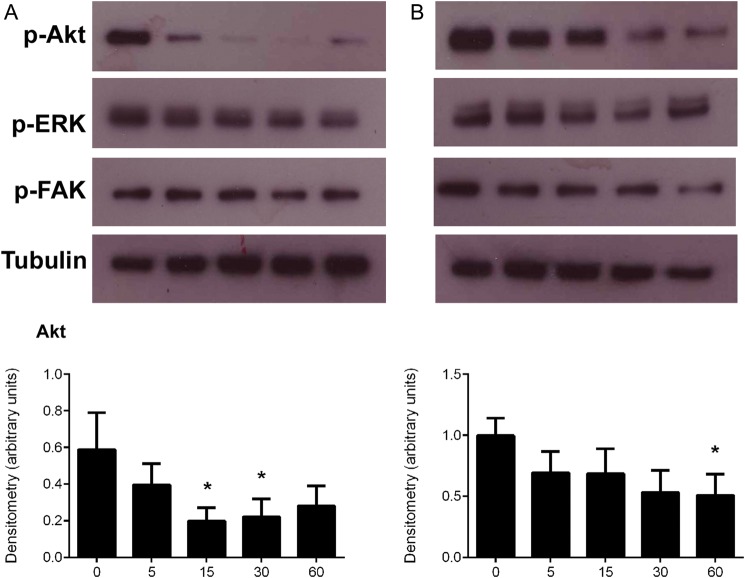
Signalling pathways affected in SGHPL-4 cells by recombinant human endostatin and recombinant human angiogenin. (**A**) SGHPL-4 cells were cultured in the presence of 500 ng/ml recombinant endostatin for 0 to 60 min. Cell lysates were collected, and equal amounts of total protein were subjected to western blot analysis for the phosphorylation of phosphorylated (p)-Akt, p-ERK 1/2 or p-FAK. Data presented are p-Akt relative to the α-tubulin loading control, mean ± SEM, *P < 0.05. (**B**) SGHPL-4 cells were cultured in the presence of 1000 ng/ml recombinant angiogenin for 0 to 60 min. Cell lysates were collected, and equal amounts of total protein were subjected to western blot analysis for phosphorylated Akt, ERK 1/2 and FAK. Data presented are p-Akt relative to the α-tubulin loading control, mean ± SEM, *P < 0.05, *n* = 4.

To identify a mechanism for the effects of endostatin and angiogenin on SGHPL-4 cells, we investigated signalling pathways known to be activated by these proteins and important in motility and invasion of trophoblast cells (Kim *et al.*, 2002; Pollheimer *et al.*, 2011; Grant *et al.*, 2012). SGHPL-4 cells were incubated for 0–60 min with recombinant 500 ng/ml endostatin or 1000 ng/ml angiogenin, concentrations previously found to influence trophoblast behaviour, and the signalling pathways activated were examined by western blot analysis. Endostatin led to a temporary de-phosphorylation of Akt^ser473^ at 15 and 30 min (Fig. 4A, 15 min: 2.95-fold, 30 min: 2.65-fold, *P* < 0.05), however, phosphorylation of ERK 1/2 and FAK was not significantly altered at any time point. Angiogenin significantly decreased phosphorylation of Akt^ser473^ at 60 min (Fig. 4B, 1.9 ± 0.17-fold decrease, *P* < 0.05), however, did not significantly affect ERK1/2 phosphorylation or phosphorylation of phopsho-FAK at any time point.

## Discussion

The major maternal immune cell component of the maternal decidua is made up of dNK cells, but the role that they play in controlling trophoblast function is still unknown. Decidual NK cells were isolated from first trimester termination of pregnancy samples, and separated into two groups based on uterine artery Doppler resistance index, of which a high-RI is a proxy measure of the extent of poor spiral artery remodelling. We examined the profile of cytokine and angiogenic factor secretion in the culture media of dNK cells from high-RI and normal-RI pregnancies. Angiogenin and endostatin were produced at a higher level by dNK cells from high-RI pregnancies, and endostatin was found to inhibit trophoblast invasion and endothelial-like trophoblast tube formation, while angiogenin was found to inhibit trophoblast invasion but promote tube formation.

In our initial screen, no difference was found between normal-RI and high-RI dNK cell secretion of a number of factors. With the exception of IL-1RA, these have all previously been confirmed as produced by dNK cells (Wallace *et al.*, 2012). The angiogenic factors and cytokines secreted by dNK cells at the maternal–fetal interface are thought to have crucial roles in controlling trophoblast functions prior to the remodelling of spiral arteries. For example, dNK are capable of chemoattraction of EVT via IL-8 and interferon induced-protein 10 (IP-10; Hanna *et al.*, 2006). Decidual NK secreted cytokines can also alter the differentiation of EVT into the invasive phenotype (Jovanovic *et al.*, 2010) and support the development of EVT into ‘endothelial-like’ structures through secreted factors including VEGF (Hu *et al.*, 2010).

Four factors were found at significantly different concentrations in the culture media of our two groups of dNK cells: sIL-2R, PLGF, endostatin and angiogenin. The concentration of soluble IL-2R was increased in the culture supernatant of dNK cells from high-RI pregnancies. Secretion of this cytokine is often preceded by increased cell-surface expression of IL-2R (Treiber-Held *et al.*, 1996), which can occur due to increased IL-2 and IL-15 signalling, two cytokines present at the maternal–fetal interface (Verma *et al.*, 2000; Scaife *et al.*, 2006). This may suggest an elevated activation status of dNK cells from high-RI pregnancies, which would also indicate altered proliferation and survival (King *et al.*, 1999). It is therefore an important future study to investigate the activation status of dNK cell-associated IL-2R in the normal- and high-RI populations, in combination with the receptor repertoire of dNK cells in each group.

The angiogenic factor PLGF was also secreted at increased levels by dNK cells from high-RI pregnancies. PLGF at the maternal–fetal interface has been shown to have both pro- and anti-angiogenic effects via the receptors it shares with VEGF, VEGFR1 and the fms-like tyrosine kinase-1 (flt-1) receptor (Furuya *et al.*, 2011). Altered PLGF expression has been associated with pre-eclampsia (Anderson *et al.*, 2012). PLGF promotes trophoblast proliferation (Athanassiades and Lala, 1998) and survival (Desai *et al.*, 1999), however, the role of dNK cell secreted PLGF may be difficult to determine as the interaction between PLGF, VEGF and flt-1 may be more important in the function of PLGF as opposed to the concentration of PLGF alone (Furuya *et al.*, 2011).

The functions of endostatin and angiogenin are unknown in the decidua. Endostatin is the C-terminal cleavage product of Collagen XVIII, an extracellular matrix protein associated with the basement membrane. The endostatin present in the dNK culture medium may therefore be a result of dNK produced proteases cleaving collagen produced by other cells in our cultures, or any bound extracellular matrix still present after dNK isolation, which could indicate higher protease levels in the high-RI dNK group. Endostatin is an anti-angiogenic factor in cancer, and most likely works through several modes of action including direct interactions with the basement membrane, inhibition of MMPs and interaction with endothelial cell receptors (Karamouzis and Moschos, 2009). Endostatin has been shown to be expressed in the decidua (Pollheimer *et al.*, 2004) and cultured endometrial stromal cells (Nasu *et al.*, 2004). Endostatin has been demonstrated to reduce invasion of the trophoblast cell line SGHPL-5 as well as the outgrowth of EVT from placental villous explants (Pollheimer *et al.*, 2011). In the present study, we determined that endostatin reduced EGF-stimulated trophoblast invasion and reduced the basal capacity of SGHPL-4 cells to form endothelial-like tubes, and may function in SGHPL-4 cells by inhibition of phosphorylation of Akt^ser473^. However, it is likely that endostatin is also able to function by alternative signalling, for example endostatin can interact with α_v_ integrins to alter cell migration and survival (Rehn *et al.*, 2001), and expression of the same integrin receptors on trophoblast are key to their invasion (Jovanovic *et al.*, 2010) and the adoption of an endothelial-like phenotype such as the endovascular trophoblast (Zhou *et al.*, 1997).

The expression of angiogenin has been demonstrated by dNK cells (Engert *et al.*, 2007), trophoblast (Rajashekhar *et al.*, 2005) and decidual stromal cells (Koga *et al.*, 2001). It is a pro-angiogenic secreted ribonuclease that binds to an unknown receptor leading to endocytosis and pro-angiogenic effects in the cell nuclei, including proliferation (Moroianu and Riordan, 1994). Despite the known role of angiogenin in vessel remodelling, its actions during pregnancy on spiral arteries or trophoblast are unknown. In our invasion assay, angiogenin inhibited, rather than promoted, EGF-stimulated SGHPL-4 invasion. However, the formation of endothelial-like trophoblast tubes was promoted by angiogenin, indicating that differentiation into endovascular trophoblast would be promoted by this factor. We examined three signalling pathways known to be involved in angiogenin or SGHPL-4 signal transduction, including phosphorylation of Akt^ser473^, ERK 1/2 and FAK (Gao and Xu, 2008; LaMarca *et al.*, 2008; Wei *et al.*, 2011) and found that Akt^ser473^ phosphorylation was time-dependently decreased by angiogenin. It is possible that the increased levels of angiogenin in the high-RI dNK group may contribute during pregnancy towards preventing vessel breakdown in the initial stages of spiral artery remodelling and maintaining vessel stability rather than direct effects on trophoblast.

The role of dNK cell-secreted factors during the first trimester of pregnancy is now recognized to be important in signalling not only to invasive trophoblast but also to decidual spiral arteries and during remodelling of the decidua (Smith *et al.*, 2009; Fraser *et al.*, 2012). Dissecting the role of dNK cells is complex because of the number of cytokines and angiogenic factors produced by these cells, some of which can have opposing effects, as evidenced by the inhibition of tube formation by endostatin and promotion by angiogenin. The overall outcome in the decidua will depend on the balance of these interactions, and also the concentration of these factors; the concentrations of these secreted factors in the decidua are unknown, and we have therefore used a range of concentrations commonly used in the literature to examine both angiogenin and endostatin. However, it is important to highlight that the interpretation of these results may be altered in the context of the correct concentration of angiogenin and endostatin, as it is possible that local concentrations between cells in the decidua may be within a different range to those described here. Future functional co-culture studies may be able to tell us more about the specific effects of these dNK cell-derived factors and the implications of these effects in normal and pre-eclamptic pregnancies. Our use of Doppler ultrasound to differentiate between pregnancies most and least likely to display poor spiral artery remodelling will further aid the discovery of altered signalling in pathological pregnancies.

## Authors' roles

R.F., A.E.W., A.P.J., G.S.W and J.E.C. conceived all of the experiments. A.E.W., R.F., J.E.C., S.G. and S.S.G. carried out all of the experiments. The manuscript was prepared by A.E.W., R.F. and J.E.C. and all authors critically revised the manuscript and approved the final version.

## Funding

This study was supported by the Wellcome Trust (project reference 091550). R.F. was a recipient of a PhD studentship from the Division of Biomedical Sciences, St. George's, University of London. Funding to pay the Open Access publication charges for this article was provided by The Wellcome Trust.

## Conflict of interest

No conflicts of interest are declared.

## References

[DEU017C1] Anderson UD, Olsson MG, Kristensen KH, Akerstrom B, Hansson SR (2012). Review: biochemical markers to predict preeclampsia. Placenta.

[DEU017C2] Ashton SV, Whitley GS, Dash PR, Wareing M, Crocker IP, Baker PN, Cartwright JE (2005). Uterine spiral artery remodeling involves endothelial apoptosis induced by extravillous trophoblasts through Fas/FasL interactions. Arterioscler Thromb Vasc Biol.

[DEU017C3] Athanassiades A, Lala PK (1998). Role of placenta growth factor (PIGF) in human extravillous trophoblast proliferation, migration and invasiveness. Placenta.

[DEU017C4] Brosens IA, Robertson WB, Dixon HG (1970). The role of the spiral arteries in the pathogenesis of pre-eclampsia. J Pathol.

[DEU017C5] Cartwright JE, Holden DP, Whitley GS (1999). Hepatocyte growth factor regulates human trophoblast motility and invasion: a role for nitric oxide. Br J Pharmacol.

[DEU017C6] Choy MY, Manyonda IT (1998). The phagocytic activity of human first trimester extravillous trophoblast. Hum Reprod.

[DEU017C7] Desai J, Holt-Shore V, Torry RJ, Caudle MR, Torry DS (1999). Signal transduction and biological function of placenta growth factor in primary human trophoblast. Biol Reprod.

[DEU017C8] Engert S, Rieger L, Kapp M, Becker JC, Dietl J, Kammerer U (2007). Profiling chemokines, cytokines and growth factors in human early pregnancy decidua by protein array. Am J Reprod Immunol.

[DEU017C9] Fraser R, Whitley GS, Johnstone AP, Host AJ, Sebire NJ, Thilaganathan B, Cartwright JE (2012). Impaired decidual natural killer cell regulation of vascular remodelling in early human pregnancies with high uterine artery resistance. J Pathol.

[DEU017C10] Furuya M, Kurasawa K, Nagahama K, Kawachi K, Nozawa A, Takahashi T, Aoki I (2011). Disrupted balance of angiogenic and antiangiogenic signalings in preeclampsia. J Pregnancy.

[DEU017C11] Gao X, Xu Z (2008). Mechanisms of action of angiogenin. Acta Biochim Biophys Sin.

[DEU017C12] Grant I, Cartwright JE, Lumicisi B, Wallace AE, Whitley GS (2012). Caffeine inhibits EGF-stimulated trophoblast cell motility through the inhibition of mTORC2 and Akt. Endocrinology.

[DEU017C13] Hanna J, Goldman-Wohl D, Hamani Y, Avraham I, Greenfield C, Natanson-Yaron S, Prus D, Cohen-Daniel L, Arnon TI, Manaster I (2006). Decidual NK cells regulate key developmental processes at the human fetal-maternal interface. Nat Med.

[DEU017C14] Harris LK, Smith SD, Keogh RJ, Jones RL, Baker PN, Knofler M, Cartwright JE, Whitley GS, Aplin JD (2010). Trophoblast- and vascular smooth muscle cell-derived MMP-12 mediates elastolysis during uterine spiral artery remodeling. Am J Pathol.

[DEU017C16] Hu Y, Eastabrook G, Tan R, MacCalman CD, Dutz JP, von Dadelszen P (2010). Decidual NK cell-derived conditioned medium enhances capillary tube and network organization in an extravillous cytotrophoblast cell line. Placenta.

[DEU017C17] Jovanovic M, Stefanoska I, Radojcic L, Vicovac L (2010). Interleukin-8 (CXCL8) stimulates trophoblast cell migration and invasion by increasing levels of matrix metalloproteinase (MMP)2 and MMP9 and integrins alpha5 and beta1. Reproduction.

[DEU017C18] Karamouzis MV, Moschos SJ (2009). The use of endostatin in the treatment of solid tumors. Expert Opin Biol Ther.

[DEU017C19] Keogh RJ, Harris LK, Freeman A, Baker PN, Aplin JD, Whitley GS, Cartwright JE (2007). Fetal-derived trophoblast use the apoptotic cytokine tumor necrosis factor-alpha-related apoptosis-inducing ligand to induce smooth muscle cell death. Circ Res.

[DEU017C20] Kim YM, Hwang S, Pyun BJ, Kim TY, Lee ST, Gho YS, Kwon YG (2002). Endostatin blocks vascular endothelial growth factor-mediated signaling via direct interaction with KDR/Flk-1. J Biol Chem.

[DEU017C21] King A, Burrows T, Verma S, Hiby S, Loke YW (1998). Human uterine lymphocytes. Hum Reprod Update.

[DEU017C22] King A, Gardner L, Loke YW (1999). Co-stimulation of human decidual natural killer cells by interleukin-2 and stromal cells. Hum Reprod.

[DEU017C23] Koga K, Osuga Y, Tsutsumi O, Yano T, Yoshino O, Takai Y, Matsumi H, Hiroi H, Kugu K, Momoeda M (2001). Demonstration of angiogenin in human endometrium and its enhanced expression in endometrial tissues in the secretory phase and the decidua. J Clin Endocrinol Metab.

[DEU017C24] LaMarca HL, Dash PR, Vishnuthevan K, Harvey E, Sullivan DE, Morris CA, Whitley GS (2008). Epidermal growth factor-stimulated extravillous cytotrophoblast motility is mediated by the activation of PI3-K, Akt and both p38 and p42/44 mitogen-activated protein kinases. Hum Reprod.

[DEU017C25] Lash GE, Schiessl B, Kirkley M, Innes BA, Cooper A, Searle RF, Robson SC, Bulmer JN (2006). Expression of angiogenic growth factors by uterine natural killer cells during early pregnancy. J Leukoc Biol.

[DEU017C26] Meekins JW, Pijnenborg R, Hanssens M, McFadyen IR, van Asshe A (1994). A study of placental bed spiral arteries and trophoblast invasion in normal and severe pre-eclamptic pregnancies. Br J Obstet Gynaecol.

[DEU017C27] Melchiorre K, Wormald B, Leslie K, Bhide A, Thilaganathan B (2008). First-trimester uterine artery Doppler indices in term and preterm pre-eclampsia. Ultrasound Obstet Gynecol.

[DEU017C28] Moroianu J, Riordan JF (1994). Nuclear translocation of angiogenin in proliferating endothelial cells is essential to its angiogenic activity. Proc Natl Acad Sci USA.

[DEU017C29] Murphy SP, Tayade C, Ashkar AA, Hatta K, Zhang J, Croy BA (2009). Interferon gamma in successful pregnancies. Biol Reprod.

[DEU017C30] Nasu K, Nishida M, Fukuda J, Kawano Y, Nishida Y, Miyakawa I (2004). Hypoxia simultaneously inhibits endostatin production and stimulates vascular endothelial growth factor production by cultured human endometrial stromal cells. Fertil Steril.

[DEU017C31] Pijnenborg R, Anthony J, Davey DA, Rees A, Tiltman A, Vercruysse L, van Assche A (1991). Placental bed spiral arteries in the hypertensive disorders of pregnancy. Br J Obstet Gynaecol.

[DEU017C32] Pijnenborg R, Vercruysse L, Hanssens M (2006). The uterine spiral arteries in human pregnancy: facts and controversies. Placenta.

[DEU017C33] Pollheimer J, Bauer S, Huber A, Husslein P, Aplin JD, Knofler M (2004). Expression pattern of collagen XVIII and its cleavage product, the angiogenesis inhibitor endostatin, at the fetal–maternal interface. Placenta.

[DEU017C34] Pollheimer J, Haslinger P, Fock V, Prast J, Saleh L, Biadasiewicz K, Jetne-Edelmann R, Haraldsen G, Haider S, Hirtenlehner-Ferber K (2011). Endostatin suppresses IGF-II-mediated signaling and invasion of human extravillous trophoblasts. Endocrinology.

[DEU017C35] Prefumo F, Sebire NJ, Thilaganathan B (2004). Decreased endovascular trophoblast invasion in first trimester pregnancies with high-resistance uterine artery Doppler indices. Hum Reprod.

[DEU017C36] Prefumo F, Fratelli N, Ganapathy R, Bhide A, Frusca T, Thilaganathan B (2008). First trimester uterine artery Doppler in women with previous pre-eclampsia. Acta Obstet Gynecol Scand.

[DEU017C37] Rajashekhar G, Loganath A, Roy AC, Chong SS, Wong YC (2005). Hypoxia up-regulated angiogenin and down-regulated vascular cell adhesion molecule-1 expression and secretion in human placental trophoblasts. J Soc Gynecol Investig.

[DEU017C38] Rehn M, Veikkola T, Kukk-Valdre E, Nakamura H, Ilmonen M, Lombardo C, Pihlajaniemi T, Alitalo K, Vuori K (2001). Interaction of endostatin with integrins implicated in angiogenesis. Proc Natl Acad Sci USA.

[DEU017C39] Santoni A, Zingoni A, Cerboni C, Gismondi A (2007). Natural killer (NK) cells from killers to regulators: distinct features between peripheral blood and decidual NK cells. Am J Reprod Immunol.

[DEU017C40] Scaife PJ, Bulmer JN, Robson SC, Innes BA, Searle RF (2006). Effector activity of decidual CD8+ T lymphocytes in early human pregnancy. Biol Reprod.

[DEU017C41] Smith SD, Dunk CE, Aplin JD, Harris LK, Jones RL (2009). Evidence for immune cell involvement in decidual spiral arteriole remodeling in early human pregnancy. Am J Pathol.

[DEU017C42] Treiber-Held S, Stewart DM, Kurman CC, Nelson DL (1996). IL-15 induces the release of soluble IL-2Ralpha from human peripheral blood mononuclear cells. Clin Immunol Immunopathol.

[DEU017C43] Verma S, Hiby SE, Loke YW, King A (2000). Human decidual natural killer cells express the receptor for and respond to the cytokine interleukin 15. Biol Reprod.

[DEU017C44] Wallace AE, Fraser R, Cartwright JE (2012). Extravillous trophoblast and decidual natural killer cells: a remodelling partnership. Hum Reprod Update.

[DEU017C45] Wallace AE, Host AJ, Whitley GS, Cartwright JE (2013). Decidual natural killer cell interactions with trophoblasts are impaired in pregnancies at an increased risk of preeclampsia. Am J Pathol.

[DEU017C46] Wei S, Gao X, Du J, Su J, Xu Z (2011). Angiogenin enhances cell migration by regulating stress fiber assembly and focal adhesion dynamics. PLoS One.

[DEU017C47] Zhou Y, Fisher SJ, Janatpour M, Genbacev O, Dejana E, Wheelock M, Damsky CH (1997). Human cytotrophoblasts adopt a vascular phenotype as they differentiate. A strategy for successful endovascular invasion?. J Clin Invest.

